# Ionizing radiation-induced long noncoding RNA CRYBG3 regulates YAP/TAZ through mechanotransduction

**DOI:** 10.1038/s41419-022-04650-x

**Published:** 2022-03-04

**Authors:** Lijun Zheng, Chenyu Luo, Nan Yang, Hailong Pei, Mintao Ji, Yinyin Shu, Zhisen Zhang, Shuai Dong, Xiuxiu Wang, Xuemei Li, Wensheng Zhang, Yan Wang, Bingyan Li, Tom K. Hei, Guangming Zhou, Lei Chang

**Affiliations:** 1 State Key Laboratory of Radiation Medicine and Protection, School of Radiation Medicine and Protection, Collaborative Innovation Center of Radiation Medicine of Jiangsu Higher Education Institutions, Medical College of Soochow University, Suzhou, China; 2 Binzhou Medical University, Yantai, China; 3 The Laboratory Animal Center of Soochow University, Suzhou, China; 4 Department of Nutrition and Food Hygiene, Soochow University of Public Health, Suzhou, China; 5 Center for Radiological Research, College of Physician and Surgeons, Columbia University Medical Center, New York, NY USA

**Keywords:** Oncogenes, Long non-coding RNAs, Stress fibres

## Abstract

Mechanotransduction sensing of tissue architecture and cellular microenvironment is a fundamental regulator of cell fate, including cancer. Meanwhile, long noncoding RNAs (lncRNAs) play multifunctions during cancer development and treatment. However, the link between lncRNAs and cellular mechanotransduction in the context of cancer progression has not yet been elucidated. In this study, using atomic force microscopy (AFM), we find that ionizing radiation reduces tumor stiffness. Ionizing radiation-induced lncRNA CRYBG3 can blunt YAP/TAZ activity through interference with mechanotransduction, resulting in the inhibition of cell proliferation, invasion, and metastasis of lung cancer cells. In vivo, we found that loss of lncRNA CRYBG3 could power the tumor initiation and metastasis ability, but this was abolished by concomitant deplete TAZ. At the molecular level, lncRNA CRYBG3 that in turn dysregulates F-actin organization, activates the LATS1/2 kinase, all in all resulting in YAP/TAZ nuclear exclusion. Our research proposes that lncRNA CRYBG3 is a mediator of radiotherapy through its control of cancer-tissue mechanotransduction and wiring YAP/TAZ activity to control tumor growth and metastasis.

## Introduction

Solid tumor cells are surrounded by a heterogeneous array of nontransformed stromal cells and extracellular matrix (ECM). Cells receive mechanical signals from cell–cell and cell–ECM interplay [1, 2]. Mechanical input can affect cell behavior, change cell fate into an oncogenic path, or control the cancer cell’s metastatic potential [3–6]. Cells receive physical–mechanical signals through mechanical receptors on the membrane surface, which trigger physical and chemical changes inside the cells that ultimately affect cytoskeletal structure [7–9]. Recently, the transcriptional coactivators Yes-associated protein (YAP), and its paralog transcriptional coactivator with PDZ-binding motif (TAZ, also known as WWTR1), have emerged as essential sensors through which cells read cytoskeletal structural and mechanical features of their surrounding microenvironment by mechanotransduction [10, 11].

YAP/TAZ can regulate cell proliferation, invasion, and metastasis in response to a wide range of extracellular and intracellular signals, including cell–cell contact, cell polarity, and mechanical cues [12–14]. YAP/TAZ are regulated by the Hippo cascade, which leads to YAP/TAZ serine/threonine phosphorylation, which changes their stability and cellular localization [10, 15, 16]. Another critical layer of YAP/TAZ regulation level is mechanotransduction, the organization and integrity of the F-actin cytoskeleton that appears to integrate both Hippo-dependent and -independent inputs [17, 18]. For example, Cofilin, CapZ, and Gelsolin, which operate as actin-capping and/or -severing proteins, control YAP/TAZ activity through F-actin organization [19].

Radiotherapy is an effective cancer-treatment modality treating over 65% of all cancer patients either as a single agent or in combination with either surgery or chemotherapy [20]. Radiation causes DNA damages to cancer cells and results in cell death [21, 22]. It has recently been found that radiotherapy induces many other biological effects that ultimately synergize to kill cancer cells [20, 23]. For example, there is evidence that radiation fuels the immune system to clear cancer cells [24, 25], as well as to change the cancer cell microenvironment to influence cell survival [26, 27]. However, there are no pioneer studies to value the tumor’s mechanostatutes after radiotherapy. In this study, we systemically examined the tumors’ mechanoenvironment changes after IR. At the molecular level, we discovered that ionizing radiation-induced lncRNA CRYBG3 impairs F-actin organization. Impeding the mechanotransduction pathway, activates Hippo signaling, thus blunting YAP/TAZ nuclear localization and transcriptional activity, thereby restricting tumor growth and metastatic potential.

## Materials and methods

### Cell culture

Human lung cancer cells A549 and Calu-1 were obtained from the American Type Culture Collection (ATCC) and were cultured in RPMI-1640 medium (Gibco) supplemented with 10% fetal bovine serum (FBS) and 1% penicillin/streptomycin. HEK293GP cells were obtained from ATCC and cultured in DMEM medium (Gibco) supplemented with 10% FBS and 1% penicillin/streptomycin. All cell lines were cultured, maintained, and used within 10–20 passages according to the requirements.

### Plasmids and reagents

Reagents were purchased as follows: Blasticidin was obtained from InvivoGen. Puromycin was obtained from Solarbio. Matrigel was obtained from Corning. Human TAZ shRNA plasmid used as reported in [28], 8 × GTIIC-Lux vector (Addgene plasmid # 34615).

### Lenti- and retrovirus preparation

Human TAZ shRNA retroviral particles were prepared by transiently transfecting HEK293GP with retroviral vectors (15 μg per 55-cm2 dish) together with pMD2-Env (5 μg per 55-cm2 dish) using Lipofectamine 2000 (Invitrogen). Then human TAZ shRNA retroviral particles were transfected and subsequently screened with a medium containing 10 μg/mL blasticidin and double-checked with RT-PCR.

*LncRNA CRYBG3* shRNA lentivirus particles (Sangon, China) were transfected into cells and subsequently screened with a medium containing 2 μg/mL puromycin and double-checked with RT-PCR. LNC CRYBG3 adenovirus particles (Sangon, China) were used for lncRNA CRYBG3 overexpression.

### Western blot

Cells were lysed using lysis buffer (50 mM HEPES (pH 7.5), 100 mM NaCl, 50 mM KCl, 1% Triton X-100, 5% glycerol, 0.5% NP-40, 2 mM MgCl2, 1 μM DTT, cocktail, and PMSF) and then followed by sonication and centrifugation at 4 °C. Extracts were quantified using the BCA method. Proteins were run on 4–12% SurePAGE-MOPS acrylamide gels (Genscript, China) and transferred onto PVDF membranes. Blots were blocked with 0.5% nonfat dry milk and incubated overnight at 4 °C with primary antibodies. Secondary antibodies were incubated for 1.5 h at room temperature, and then blots were developed with chemiluminescent reagents. Images were acquired with FluroChem M1 (Protein Simple).

For cell-component separation assay, nuclei of A549 cells were isolated from confluent A549 cells grown on 10-cm dishes by hypotonic lysis in 5 ml buffer 1 (20 mM HEPES (pH 7.5), 10 mM KCl, 0.1% NP-40, 5% glycerol, 5 mM MgCl2, 1 μM DTT, and phosphatase and protease inhibitors) for 5 min. After centrifugation at 600 g for 3 min, the supernatant was saved, whereas the nuclear pellet was resuspended in lysis buffer for western blot analysis.

The antibodies used for western blot were anti-YAP/TAZ (sc-101199) was from Santa Cruz. Antiphosphorylated YAP (S127) (CST 4911), anti-LATS1 (CST 3477), anti-phosphorylated LATS (Thr1079) (CST 8654), and anti-PCNA (CST 13110) were from Cell Signaling Technology. Anti-GAPDH (3241215) was from Millipore.

### Quantitative real-time PCR (qRT-PCR)

Cells were collected using the BioFlux Kit (Bioer, China) for total RNA extraction. Total RNA was extracted from tumor tissues by using TriZOL Reagent (Ambion, USA). qRT-PCR analyses were carried out on reverse-transcribed cDNAs with QuantStudio 1 (Applied Biosystems, ThermoFisher Scientific) and analyzed with QuantStudio Design & Analysis Software (version 1.5.1). Expression levels are always normalized to GAPDH. The primers are used as reported [19, 29, 30].

### RNA interference

siRNA transfections were done with Lipofectamine RNAi-MAX (Thermo Fisher Scientific) in antibiotics-free medium according to the manufacturer’s instructions. The primer sequences are used as reported [19, 29, 30].

### Immunofluorescence

Cells cultured on coverslips were fixed with 4% paraformaldehyde for 30 min. The cultures were then washed 3 times with PBS, permeabilized with 0.5% Triton X-100 in PBS for 10 min, blocked with 10% BSA and 0.1% Triton X-100 in PBS for 1 h, incubated with primary antibodies (overnight, 4°C), and then washed 3 times with PBS followed by incubation with secondary antibodies (1.5 h, room temperature). Phalloidin was added after the secondary antibodies for 15 min, whereas ProLong-DAPI was added after secondary antibodies or Phalloidin for 15 min. Images were acquired using a confocal microscope (FV1200, Olympus). More than 5 fields of view from at least three independent experiments were randomly chosen.

Primary antibodies were anti-YAP/TAZ (sc-101199) was from Santa Cruz. Anti-phospho-Myosin Light Chain 2 (p-MLC2) (CST 3675) was from Cell Signaling Technology. Secondary antibodies were from Beyotime. ProLong-DAPI was from Invitrogen. Phalloidin (PHDH1) was from Cytoskeleton.

### Immunohistochemistry

Xenografted tumor tissues were sacrificed and embedded in OCT and then rapidly frozen. Cryostat sections were cut and dried on glass slides at room temperature. Then cryostat sections were fixed with tissue-fixation fluid for 30 min. Endogenous peroxidase blocking was performed by adding 1–2 drops of 3% hydrogen peroxidase, enough to cover the sections, followed by incubation for 10 min. It was permeabilized with 0.5% Triton X-100 in PBS for 10 min, blocked with 5% BSA and 0.1% Triton X-100 in PBS for 1 h, incubated with primary antibodies (2 h, room temperature), and then washed 3 times with PBS, incubated with secondary antibodies (0.5 h, room temperature). Immunoreactions were visualized using 3,3′-diaminobenzidine tetrahydrochloride hydrate (DAB) with subsequent counterstaining with Mayer’s hematoxylin using an inverted microscope (Leica). The antibody anti-Ki-67 (ab 15580) was from Abcam.

### Atomic force microscopy measurements

Atomic force microscopy (AFM) and analysis were performed to detect cell or tissue sample stiffness. AFM settings and tissue-sample preparation were optimized using cryopreserved tumor tissues. Snap-frozen tissue blocks were cut into 20-mm-thick sections. Before AFM measurements, each section was immersed in PBS and thawed at RT. Samples were maintained in proteinase inhibitor (Roche, Germany, cOmplete) in PBS during the AFM session. AFM indentations were analyzed using a Bruker mounted on an Olympus-inverted microscope. Briefly, we used silicon nitride cantilevers with a spring constant of 0.03 N/m (Bruker, USA, MLCT) and attached a polystyrene spherical ball of 10 mm in diameter (Macklin, China) using epoxy glue (Pattex, China). Cantilevers were calibrated using the thermal oscillation method before each experiment. Five 10 mm × 10 mm AFM force maps were typically obtained on each sample. The Hertz model was used to determine the elastic properties of the tissue. The upper 200 nm of tissue was considered for all fits. Tissue samples were assumed to be incompressible and a Poisson’s ratio of 0.5 was used in the calculation of the Young’s elastic modulus.

### Transwell migration assay

Cell-invasion assays were performed using transwell membranes coated with Matrigel (Corning). Briefly, the RPMI-1640 medium (600 μL) alone was placed in the lower chamber. A total of 1 × 10^5^ cells in 100 μL medium were seeded into the upper chamber (pore size, 8 μm). The chamber was then incubated in a 5% (v/v) CO2-humidified incubator at 37 °C for 24 h. The unmigrated cells in the upper chamber were removed with cotton swabs. The membrane was then fixed in 70% ethanol for 10 min and then stained with 0.1% crystal violet for 10 min at room temperature. The number of cells that have migrated to the lower surface of the membrane was photographed by using a Leica microscope fitted with a digital camera.

### Scratch-wound assay

Scratch assay was used to analyze cell migration. The cells were grown to full confluency in six-well plates and incubated 12 h in a serum-free medium. The cell surface was scratched by a sterile 200 μL pipette tip, washed with serum-free medium to remove detached cells from the plates. The wound gap was observed and photographed using a Leica microscope fitted with a digital camera at 0, 24, and 36 h. The width of the scratch was calculated using the ImageJ software.

### Colony-formation assay

A colony-formation assay was performed to detect cell growth. Cells were seeded at 200 cells/well in 6-well plates, and dispersed evenly by slightly shaking the dishes and cultured in a 5% (v/v) CO2-humidified incubator at 37 °C for 14 days. After being fixed with 70% ethanol for 10 min, the cells were stained with 0.1% crystal violet for 15 min before washing with tap water and air-drying. The clones with more than 50 cells were counted with an ordinary optical microscope. The clone-formation rate was calculated with the following formula: clone-forming efficiency ratio = (number of clones/number of cells inoculated) ×100%.

### Luciferase assays

Luciferase assays were performed in A549 and Calu-1 cells with the established YAP/TAZ-responsive luciferase reporter 8 × GTIIC-Lux. 8 × GTIIC-Lux reporter (800 ng per 4.5-cm^2^ well) was transfected together with CMV-β-gal (400 ng per 4.5-cm^2^ well) to normalize for transfection efficiency using a β-galactosidase Assay Kit (Beyotime, China). DNA transfections were done with TransIT-LT1 (Mirus Bio) according to the manufacturer’s instructions. A549 and Calu-1 cells were plated at 30% confluence (day 0), transfected with plasmid DNA (day 1), and then transfected with lncRNA CRYBG3 (day 2), and collected 24 h later (day 3). For experiments using siRNA-depleted cells, cells were plated at 15% confluence (day 0), transfected with the indicated siRNAs (day 1), changed to the culture medium and transfected with plasmid DNA (day 2), transfected with lncRNA CRYBG3 (day 3), and collected 24 h later (day 4).

### Animal experiment

All mice were randomly divided into experimental group and control group. All mice experiments were conducted on blinding. In total, 5 × 10^6^ cells were injected subcutaneously into the flanks of 4-week-old nude mice (Charles River, Beijing, China). In total, 5 × 10^6^ cells were injected subcutaneously into the flanks of 4-week-old nude mice (*n* = 6). Half of the mice were subjected to 100 μL lncRNA CRYBG3 adenovirus particles or an equal amount of LNC-control adenovirus particles once a week for 14 days. In total, 5 × 10^6^ cells transfected with shRNA of lncRNA CRYBG3 or lncRNA CRYBG3/TAZ were injected subcutaneously into the flanks of 4-week-old nude mice (*n* = 6). In total, 8 × 10^6^ Luciferase stable-expressing cell lines transfected with shRNA against either lncRNA CRYBG3 or lncRNA CRYBG3/TAZ were injected into the tail veins of NOD-SCID mice. (SLAC, Shanghai, China). After 40 days, mice were given D-Luciferin potassium salt (Beyotime, China), a luciferase substrate, and photographed using IVIS spectrum in vivo imaging system (PerkinElmer, USA) on day 40 for the observation of in vivo cancer cell metastasis.

Animals were maintained in the Animal Facilities of Soochow University under pathogen-free conditions. All studies involving mice were studied and operated followed by the rules of the Soochow University Institutional Animal Care and Use Committee.

### Statistical analyses

The number of biological and technical replicates and the number of animals is indicated in figure legends, main text and Methods. All data were presented as the mean ± s.d. in the figure legends and extended data figures. Statistical analysis was performed by Microsoft Excel software. Comparisons were analyzed by unpaired Student’s *t*-test, as indicated in figure legends and extended data figures. All inclusion/exclusion criteria were pre-established, and no samples or animals were excluded from the analysis. The investigators were blinded to allocation during experiments and outcome assessment.

## Results

### Ionizing radiation reduces tumor stiffness

**Fig. 1:**
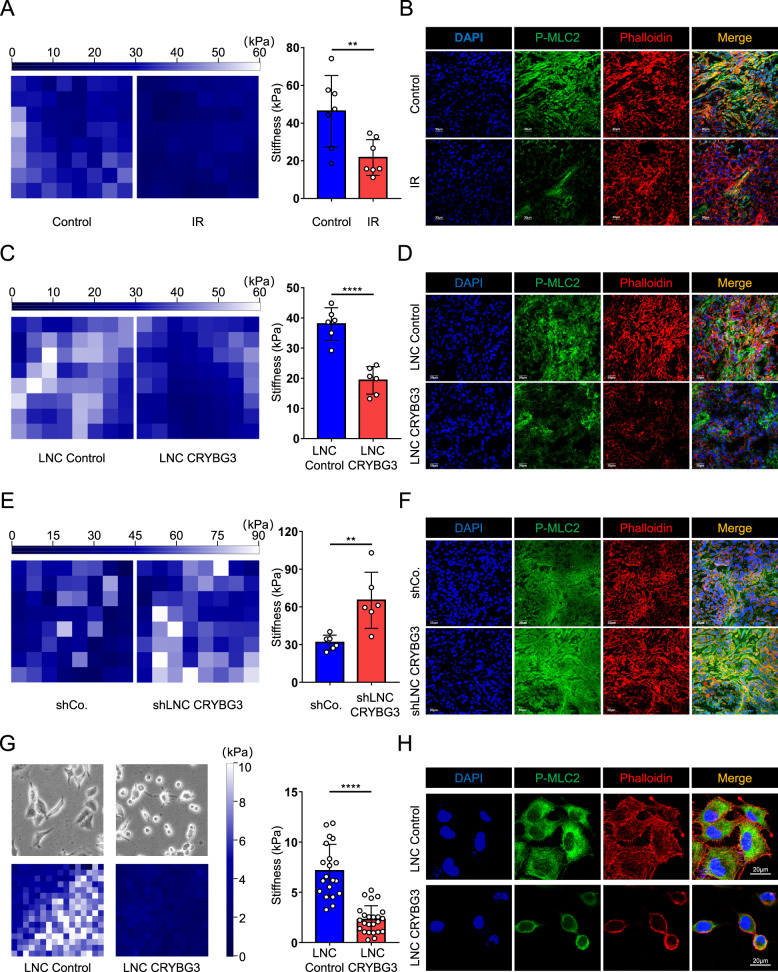
Ionizing radiation-induced lncRNA CRYBG3 regulates the stiffness of the tumor. **A** Representative atomic force microscopy stiffness-map images (left) and quantifications (right) of stiffness in xenografted tumors exposed to 10-Gy X-rays (IR group, *n* = 7) or control (control group, *n* = 7). **B** Representative immunofluorescence images of p-MLC2 (green) and phalloidin (red) in xenografted tumors exposed to 10-Gy X-rays or control. The samples were frozen, sectioned, and stained. Scale bars, 30 μm. **C** Representative stiffness-map images (left) and quantifications (right) of stiffness in xenografted tumors treated with lncRNA control (LNC Control, *n* = 6) or lncRNA CRYBG3- (LNC CRYBG3, *n* = 6) overexpressed adenovirus. **D** Representative immunofluorescence images of p-MLC2 (green) and phalloidin (red) on indicated xenografted tumors. Scale bars, 30 μm. **E** Representative stiffness-map images (left) and quantifications (right) of xenografted tumors subcutaneously injected by shRNA against lncRNA CRYBG3 (*n* = 6) or control shRNA (*n* = 6). **F** Representative immunofluorescence images of p-MLC2 (green) and phalloidin (red) on indicated xenografted tumors. Scale bars, 30 μm. **G** Representative bright-field and stiffness-map images in A549 cell-overexpressed lncRNA control or lncRNA CRYBG3. The right panels were the quantification of average Young’s modulus. **H** Representative immunofluorescence images of p-MLC2 (green) and phalloidin (red) in A549 cells. Scale bars, 20 μm. Data are presented as mean ± s.d. *P*-values were calculated by unpaired Student’s *t*-test. **p < 0.01; ****p < 0.0001.

To evaluate tumor tissues’ mechanical changes after radiotherapy, we irradiated subcutaneous tumors generated in athymic nude mice with a single, 10-Gy dose of X-rays. We then assessed the mechanical changes of xenograft tumor samples by AFM. Ionizing radiation (IR) resulted in significantly decreased tumor-tissue stiffness compared with unirradiated tumors (Fig. 1A). Collagen fibers were reduced after local irradiation, as demonstrated by Picrosirius red staining (Fig. S1A). Cells are in constant mechanical equilibrium with their environment and intercellular tension. Thus, ECM deposition is directly proportional to the inner tension and organization of the F-actin cytoskeleton. As a molecular motor that regulates actin filaments’ movement, myosin plays an essential role in maintaining the cytoskeleton’s integrity. Phosphorylated myosin light-chain 2 (p-MLC2) represents contractile actomyosin bundles and thus represents a readout of cellular mechanotransduction. In line with the above findings, IR decreased the activity of p-MLC2 in xenografted tumors (Fig. 1B). These results indicate that IR could change the mechanical property of cancer tissues in vivo.

### LncRNA CRYBG3 regulates tumor stiffness

Cellular mechanosignaling is known to foster cancer development [5, 31]. Our initial findings suggest that IR could reduce the mechanical stiffness of cancer tissue. Next, we queried the mechanism behind this phenomenon to understand how IR-induced mechanotransduction alters cell behavior. Our previous studies have found that lncRNA CRYBG3 induced by ionizing radiation can directly bind to G-actin, inhibit the formation of actin filaments, thereby exerting a tumorsuppressor function [30]. We checked lncRNA CRYBG3 expression after different types of particle radiation. The data suggest that the expression of lncRNA CRYBG3 dramatically increased after radiation (Fig. S1B). This result, together with our previous studies, led us to postulate that lncRNA CRYBG3 upregulation might be instrumental in decreasing the stiffness of tumor tissues following IR treatment.

Thus, we explored if the increased lncRNA CRYBG3 expression might have a softening effect on tumor cell plasticity. To simulate the effects of radiation, we raised lncRNA CRYBG3 by injecting lncRNA CRYBG3 (LNC CRYBG3)-overexpressing adenovirus into subcutaneous xenograft and used AFM to monitor the stiffness of lncRNA CRYBG3-overexpressing xenograft and lncRNA control xenograft samples. We found that lncRNA CRYBG3 overexpression decreased tumor-tissue stiffness (Fig. 1C). Moreover, overexpression of lncRNA CRYBG3 in tumor tissues also impeded the phosphorylation of MLC2, a marker of tissue contractility and cellular mechanotransduction (Fig. 1D). Conversely, we hypothesized that depletion of lncRNA CRYBG3 might lead to increased tumor stiffness. We subcutaneously inoculated nude mice with cells that are stably expressing short-hairpin RNA (shRNA) of lncRNA CRYBG3 or short-hairpin RNA control. LncRNA CRYBG3-deficient xenograft revealed an increase in tumor stiffness (Fig. 1E). Consistently, we found that lncRNA CRYBG3 depletion enhanced cellular tension as visualized by p-MLC2 (Fig. 1F). We also checked the stiffness of single lung cancer cells, which were cultured in vitro after overexpressing of lncRNA CRYBG3. The data show that lncRNA CRYBG3 could reduce Young’s modulus of a single cancer cell, meanwhile, change cancer cells’ morphology and cytoskeleton organization inside the cell, which phenocopies the mechanical defected cells (Fig. 1G, H), such as small cell size or cells on soft ECM, dictating the cytoskeletal structure to develop low contractile forces. The above data suggest that lncRNA CRYBG3 per se could directly regulate the mechanical properties of cancer cells.

### LncRNA CRYBG3 regulates YAP/TAZ activity

**Fig. 2:**
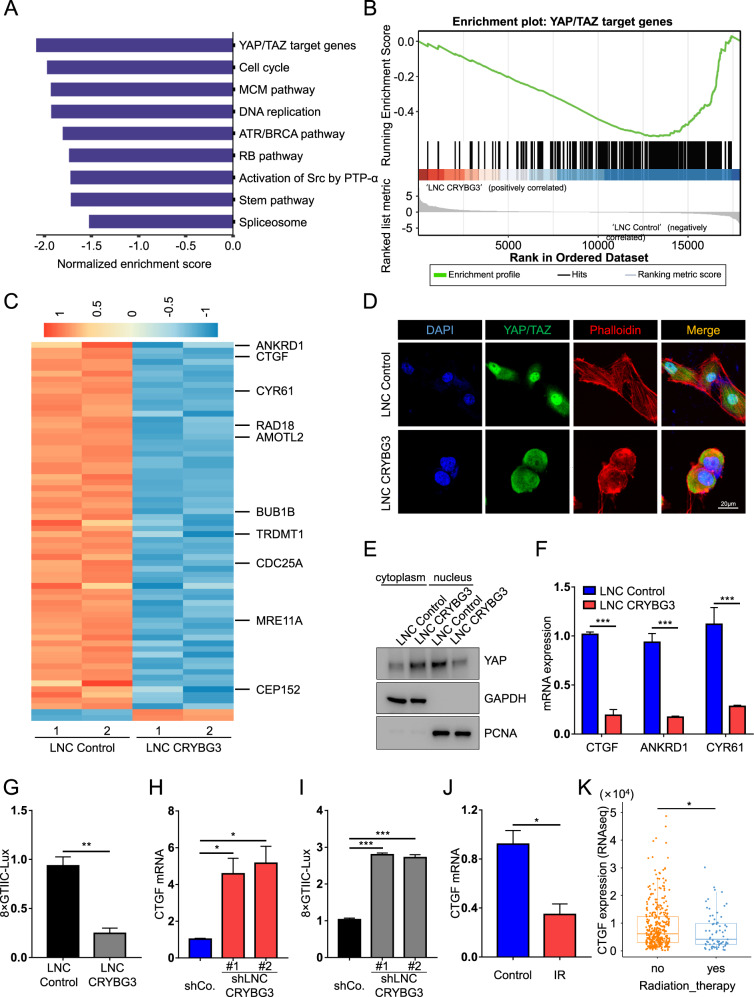
LncRNA CRYBG3 regulates YAP/TAZ activity. **A** Gene-set enrichment analysis showing the most enriched signaling pathways by normalized enrichment score in overexpressed lncRNA CRYBG3 (LNC CRYBG3) A549 cells compared with lncRNA control (LNC Control). **B** Gene-set enrichment analysis of gain of lncRNA CRYBG3 versus lncRNA Control for the expression of YAP/TAZ direct target genes. Normalized enrichment score = ­2.086, false-discovery rate *q* = 0.004. **C** Heat map showing the significantly altered 66 direct YAP/TAZ target genes in lncRNA CRYBG3 overexpressed A549 cells compared with lncRNA control. **D** Immunofluorescence analysis determined YAP/TAZ localization and the microfilaments’ morphology in Calu-1 cells. Scale bars, 20 μm. **E** Western blot analysis of YAP proteins in the cytoplasm and nucleus of A549 cells transfected with lncRNA Control or lncRNA CRYBG3. **F** qRT-PCRs assessing the expression levels of the YAP/TAZ endogenous targets *CTGF*, *ANKRD1*, and *CYR61* in Calu-1 cells. **G** Luciferase assays in Calu-1 cells transfected with a synthetic reporter for YAP–TEAD-dependent transcription (8xGTIIC-Lux). **H** qRT-PCRs assessing the expression levels of the YAP/TAZ endogenous targets *CTGF* and *CYR61* in cells transfected with shlncRNA CRYBG3 or control shRNA. **I** Luciferase assays in cells transfected with 8 × GTIIC-Lux reporter and with the indicated shRNAs. **J** qRT-PCRs assessing the expression levels of the YAP/TAZ endogenous target *CTGF* in A549 cells exposed to 4 Gy X-rays (IR group) or control (control group). **K** Dot plot showing the CTGF expression in LUSC patients with or without radiation therapy from TCGA database. (**D**), (**E**) (**F**), (**G**), (**H**), (**I**), and (**J**) are repeated three independent times and data are presented as mean ± s.d. *P-*values were calculated by unpaired Student’s *t*-test. *p < 0.05; **p < 0.01; ***p < 0.001; ****p < 0.0001.

The transcriptional coactivators YAP and TAZ have emerged as a fundamental sensor through which cells read the architectural features of their tissue microenvironment through the mechanotransduction pathway [19, 29, 32]. First, we verified whether lncRNA CRYBG3 could affect YAP/TAZ activity. We performed global transcriptome sequencing on lncRNA CRYBG3-overexpressing A549 cells to unbiasedly determine the pathways most prominently regulated by lncRNA CRYBG3 expression. Bioinformatics analysis showed that 420 genes were upregulated and 478 genes were downregulated upon lncRNA CRYBG3 overexpression (Fig. S2A, B). Using 2922 canonical pathway gene sets from the Molecular Signatures Database, together with a validated set of 379 direct YAP/TAZ target genes that were identified previously by ChIP-seq and microarray analyses [33] as unbiased references, gene-set enrichment analysis (GSEA) revealed that YAP/TAZ target genes were the most downregulated upon lncRNA CRYBG3 overexpression (Fig. 2A, B). Of note, we found that 66 out of 379 YAP/TAZ direct target genes were differentially expressed upon ectopic lncRNA CRYBG3 expression (Fig. S2C). Intriguingly, 64 out of those 66 target genes were downregulated upon lncRNA CRYBG3 overexpression, among the top hits were several well-known YAP/TAZ targets, such as *CTGF*, *CYR61, ANKRD1*, and *AMOTL1* (Fig. 2C and Fig. S2D). These findings indicated that YAP/TAZ transcriptional activity is impeded in lncRNA CRYBG3-overexpressing cells.

YAP and TAZ show a nuclear bias and transcriptional activity under a stiff ECM environment. YAP/TAZ relocalized to the cytoplasm and inactivated when cells are plated on compliant mechanical environments (e.g., small cell size and soft ECM dictating the cytoskeletal structure to develop low contractile forces). In line with our previous studies, lncRNA CRYBG3 affected cytoskeleton organization [30] (Fig. 2D and Fig. S2E, phalloidin staining). We also find that lncRNA CRYBG3 expression relocalized YAP/TAZ into the cytoplasm both in A549 and Calu-1 cells (Fig. 2D and Fig. S2E, YAP/TAZ staining, quantification in Fig. S2F). These results were also confirmed by checking nuclear and cytosolic YAP protein levels (Fig. 2E, the original western blots are in Supplemental Material 1). In addition, lncRNA CRYBG3 blocked the expression of several direct YAP/TAZ target genes, such as *CTGF*, *CYR61*, and *ANKRD1*, consistent with our sequencing results (Fig. 2F and Fig. S2G, verification in Fig. S2H). LncRNA CRYBG3 also impaired induction of the YAP–TEAD luciferase reporter (8 × GTIIC) through the inactivation of endogenous YAP/TAZ (Fig. 2G and Fig. S2I). On the other hand, depletion of lncRNA CRYBG3 by shRNA promoted the activation of endogenous YAP/TAZ transcriptional activity (Fig. 2H and verification in Fig. S2J), as assessed by the induction of the YAP–TEAD luciferase reporter compared with the control shRNA (Fig. 2I). We also detected the changes in YAP/TAZ activity of tumor cells induced by ionizing radiation, and found that the expression of CTGF and CYR61 in A549 cells decreased after X-ray irradiation (Fig. 2J and Fig. S2K). Similarly, in samples of lung cancer patients from The Cancer Genome Atlas project (*TCGA*, https://tcga-data.nci.nih.gov/tcga/), we found that CTGF expression was significantly lower in patients who received radiotherapy than in those who did not (Fig. 2K). The above results raised the possibility that lncRNA CRYBG3 may restrain YAP/TAZ activity by inhibiting cytoskeleton organization.

### LncRNA CRYBG3 regulates tumor growth and metastasis through YAP/TAZ

**Fig. 3:**
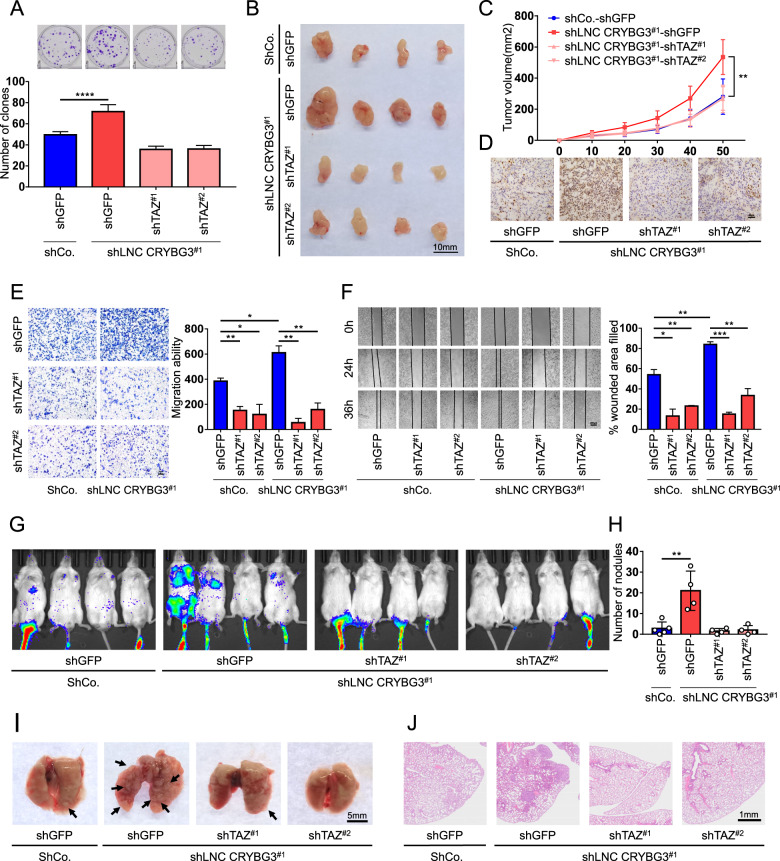
LncRNA CRYBG3 regulates tumor growth and metastasis through YAP/TAZ. **A** Representative images and quantifications of the clone-forming assay with the indicated shRNAs. **B** Image of the xenografted tumors, which were sacrificed at 50 days post injection. Scale bars, 10 mm. **C** Quantifications of the xenografted tumor-growth curve, tumor sizes were monitored every ten days. **D** Immunohistochemical images of Ki67 protein in the xenografted tumors expressed with the indicated shRNAs. Scale bars, 50 μm. **E** Transwell migration assay for cells with indicated treatment was measured for migration ability. Scale bars, 100 μm. **F** Scratch-wound assays were performed by transfection with the indicated shRNAs. At 24 h or 36 h after scratching, the percent wounded area filled was calculated. The quantifications of percent wounded area filled at 36 h. Scale bars, 200 μm. **G** In vivo metastasis assays were performed using luciferase stable-expressing cells transfected with the indicated shRNAs to be injected into the caudal vein of NOD-SCID mice, and 40 days later, images were taken by IVIS Spectrum in vivo imaging system. **H**, **I** Quantifications and representative images of the metastatic nodules on the surface of the lungs in the indicated treatment. Scale bars, 5 mm. **J** Representative HE staining images of the metastatic nodules of the lungs with indicated treatment (uncropped picture in Fig. S3D). Scale bars, 1 mm. (**A**), (**E**), and (**F**) are repeated three independent times and all data are presented as mean ± s.d. *P-*values were calculated by unpaired Student’s *t*-test. *p < 0.05; **p < 0.01; ***p < 0.001; ****p < 0.0001.

YAP and TAZ are extensively activated in human malignancies, and known to be essential in inducing cell proliferation, tumorigenesis, as well as metastasis [10, 34, 35]. We thus aimed to elucidate how the interplay between YAP/TAZ and lncRNA CRYBG3 affects malignant progression. By using shRNA-mediated silencing for the respective genes, we generated a constitutive lncRNA CRYBG3-knockdown as well as a TAZ-impaired cell lines (knock down efficiencies are verified in Fig. S3A, B). We found that knockdown of lncRNA CRYBG3 increased cells’ colony-formation ability, whereas concomitant knockdown of TAZ blunted clonogenicity (Fig. 3A), as well as YAP/TAZ activity (Fig. S3C). In vivo, constitutive knockdown of lncRNA CRYBG3 in xenograft displayed a rapid tumor growth and increased tumor volume, but this was blunted by concomitant depletion of TAZ (Fig. 3B, C). Consistently, concomitant TAZ knockdown curtailed the expansion of the Ki67-positive cell population elicited by lncRNA CRYBG3 loss (Fig. 3D). Next, we assessed whether lncRNA CRYBG3 affects tumor invasion and metastasis through YAP/TAZ. We found that loss of endogenous lncRNA CRYBG3 increased lung cancer cell invasiveness and metastatic ability, however, this was counteracted by concomitant knockdown of TAZ (Fig. 3E, F). To validate this concept in vivo, cells that constituted expressing luciferase and stably expressing shRNAs against either lncRNA CRYBG3 or both lncRNA CRYBG3/TAZ, were injected into the tail veins of NOD-SCID mice. Using the IVIS spectrum in vivo imaging system, we found that knockdown of lncRNA CRYBG3 increased the metastatic incidence in the lung, which was reverted by concomitant TAZ loss (Fig. 3G). This phenomenon was further confirmed by both gross observation and histopathological evaluation of metastatic nodules on the surface of the lung (Fig. 3H–J and Fig. S3D). These results indicated that lncRNA CRYBG3 suppresses tumor growth, lung cancer cell invasion, and metastasis by inhibiting YAP/TAZ.

### LncRNA CRYBG3 inhibits YAP/TAZ by F-actin depolymerization

**Fig. 4:**
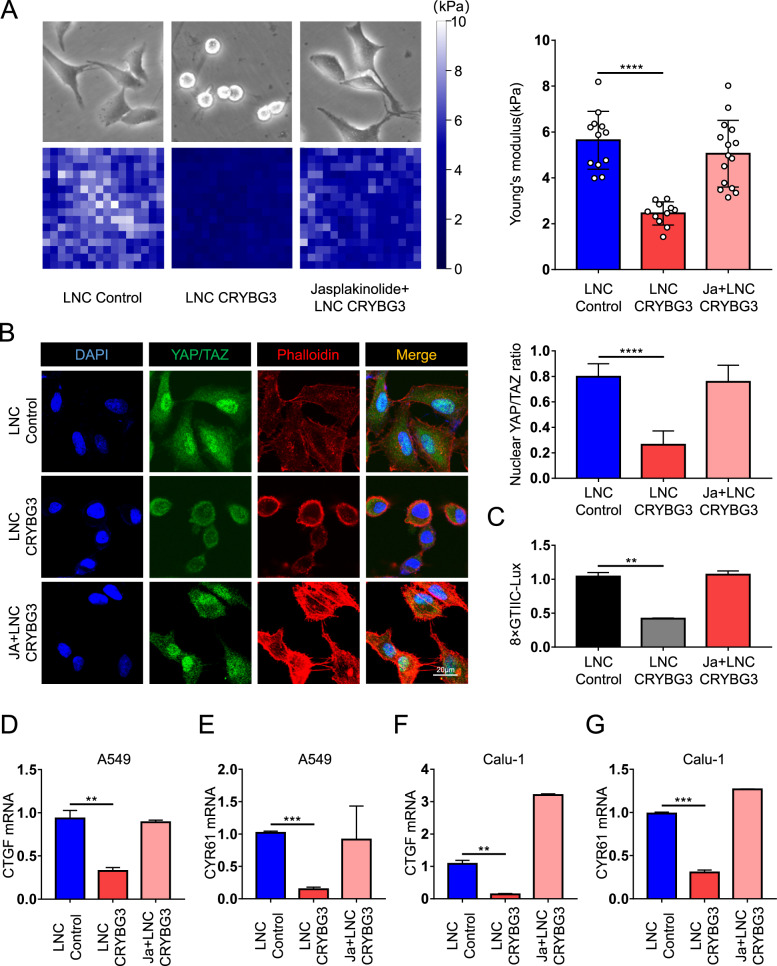
LncRNA CRYBG3 inhibits YAP/TAZ by cytoskeleton depolymerization. **A** Representative bright-field and stiffness-map images. The right panels were the quantification of average Young’s modulus. **B** Representative immunofluorescence images of YAP/TAZ (green), phalloidin (red), and quantifications of YAP/TAZ localization. Scale bars, 20 μm. **C** Luciferase assays in Calu-1 cells transfected with 8 × GTIIC-Lux reporter and as indicated treatments. **D**, **E**, **F**, and **G** qRT-PCRs assessing the expression levels of the YAP/TAZ endogenous targets *CTGF* and *CYR61* in A549 and Calu-1 cells as indicated treatments. All the experiments are repeated three independent times and data are presented as mean ± s.d. *P*-values were calculated by unpaired Student’s *t*-test. **p < 0.01; ***p < 0.001; ****p < 0.0001.

The dynamic balance between actin polymerization and depolymerization is of great significance to intracellular mechanical signaling processes. Jasplakinolide is a natural cytotoxic product that induces actin polymerization [36]. Here, we took this drug to test whether F-actin-promoting treatments could counteract the cytoskeletal destruction caused by lncRNA CRYBG3 overexpression. LncRNA CRYBG3 alone led to the depolymerization of the actin cytoskeleton, which was accompanied by morphological changes and an apparent reduction in cell size. However, concomitant treatment with jasplakinolide prevented the depolymerization of F-actin, reconstituted cell size, and rebuilt the mechanic statues of the cell (Fig. 4A, upper panel). Through atomic force microscopy, we found that jasplakinolide could restore A549 cells’ Young’s modulus, which was impaired following lncRNA CRYBG3 overexpression (Fig. 4A, lower panel). Next, we aimed to assess whether pretreatment of cells with jasplakinolide before overexpression of lncRNA CRYBG3 could restore YAP/TAZ activity. Immunofluorescence results also showed that jasplakinolide could not only restore the F-actin organization (Fig. 4B, phalloidin staining) but also relocate YAP/TAZ to the nuclear compartment even in the presence of lncRNA CRYBG3 (Fig. 4B). Using 8 × GT luciferase reporter (8 × GTIIC), we found that jasplakinolide recovered the reduced endogenous YAP/TAZ transcription activity caused by lncRNA CRYBG3 (Fig. 4C). Similarly, in both A549 and Calu-1 cells, a decrease in expression of *CTGF* and *CYR61* induced by lncRNA CRYBG3 overexpression was rescued by jasplakinolide treatment (Fig. 4D–G, verification in Fig. S4A, B). In sum, the above results suggest that lncRNA CRYBG3 depolymerizes F-actin, thereby impairing YAP/TAZ activity, which, consistently, could be rescued by the restoration of cytoskeletal structure.

### Depletion of ADF/CFL1 rescues lncRNA CRYBG3-mediated YAP/TAZ inhibition

**Fig. 5:**
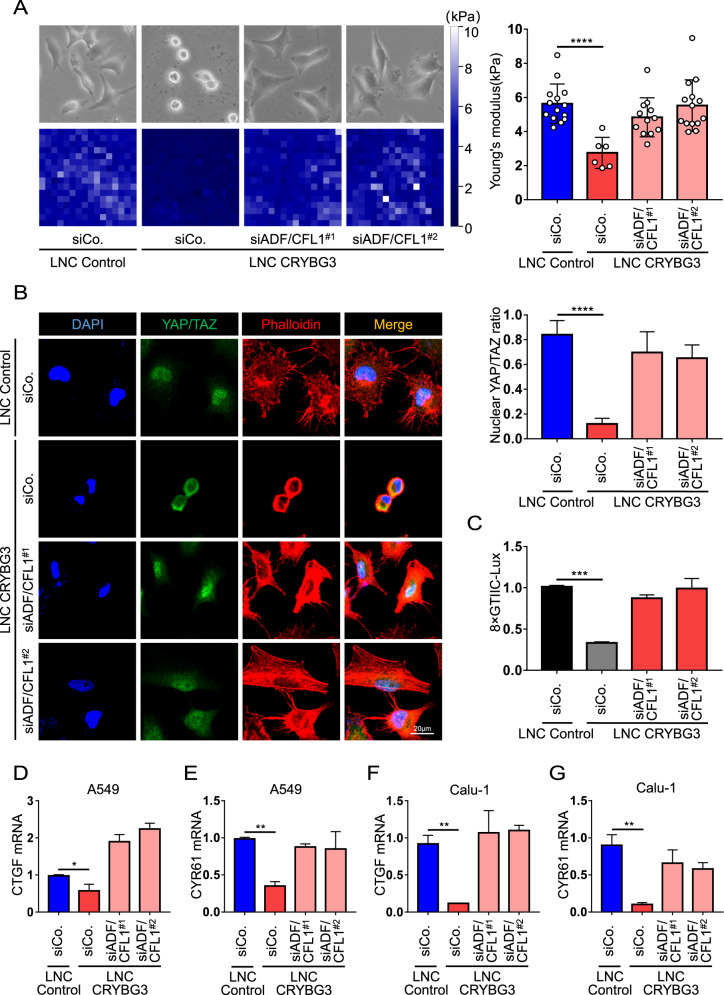
siADF/CFL1 rescue lncRNA CRYBG3-caused YAP/TAZ inhibition. **A** Representative bright-field and stiffness-map images in cell-overexpressed lncRNA control (LNC Control) or lncRNA CRYBG3 (LNC CRYBG3) after treatment with the indicated siRNA. The right panels were the quantification of average Young’s modulus. **B** Representative immunofluorescence images (left) of YAP/TAZ (green) and phalloidin (red) staining and quantifications of YAP/TAZ localization (right). Scale bars, 20 μm. **C** Luciferase assays in cells transfected with 8 × GTIIC-Lux reporter and treated as indicated. **D**, **E**, **F**, and **G** qRT-PCRs assessing the expression levels of the YAP/TAZ endogenous targets *CTGF* and *CYR61* in A549 and calu-1 cells as indicated treatments. All the experiments are repeated three independent times and data are presented as mean ± s.d. *P*-values were calculated by unpaired Student’s *t*-test. *p < 0.05; **p < 0.01; ****p < 0.0001.

The actin-depolymerizing factor/cofilin-1 (ADF/CFL1) are well-known modulators of actin-filament dynamics and, as such, has been extensively studied in eukaryotic cells [37]. It is known that these factors are involved in a variety of cellular processes, from signal transduction to the cytonuclear trafficking of actin [38]. It has been reported that ADF and cofilin-1 work as core factors in mechanotransduction in regulating YAP/TAZ activity [19]. Here, we tested if the inhibition of YAP/TAZ mechanotransduction by lncRNA CRYBG3 could be modulated by restoring F-actin structure through depletion of ADF/CFL1. Initially, we detected whether lncRNA CRYBG3 expression directly affected ADF/CFL1, and found that lncRNA CRYBG3 did not regulate the expression of ADF/CFL1 (Fig. S4C, D). However, we found that depleting ADF/CFL1 could reverse the effects on cellular morphology and Young’s modulus caused by lncRNA CRYBG3 overexpression (Fig. 5A). This result suggests that knockdown ADF/CFL1 could restore the mechanical property of cells. As visualized by phalloidin staining (Fig. 5B), depleting ADF/CFL1 counteracted the effect of the lncRNA CRYBG3 on F-actin depolymerization, which was accompanied by relocalization of YAP/TAZ into the nucleus (Fig. 5B). Next, we assessed whether siADF/CFL1 could also restore the YAP/TAZ transcription activity. Using 8 × GT luciferase reporter, depletion of ADF/CFL1 rescued lncRNA CRYBG3-mediated inhibition of YAP/TAZ transcriptional activity (Fig. 5C). Similar results were obtained in two different cell lines and with independent siRNAs (Fig. 5D–G, verification in Fig. S4E, F). These results provided further proof that regulation of actin-filament polymerization, through depletion of the F-actin depolymerizing factor ADF/CFL1, could alleviate the lncRNA CRYBG3-mediated inhibition of YAP/TAZ activity.

### LncRNA CRYBG3 regulates YAP/TAZ activity through LATS1/2 kinases

**Fig. 6:**
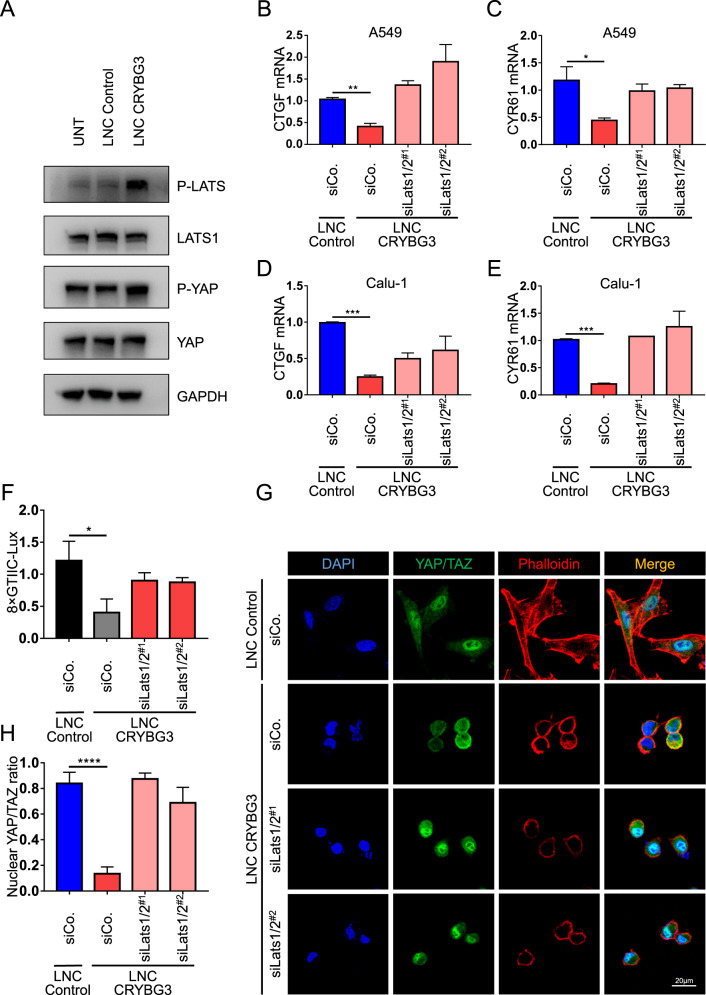
LncRNA CRYBG3 regulates YAP/TAZ activity by LATS1/2 kinase. **A** Western blot analysis of indicated proteins in A549 cells transfected with lncRNA control (LNC Control) or lncRNA CRYBG3 (LNC CRYBG3). **B**, **C**, **D**, and **E** qRT-PCRs assessing the expression levels of the YAP/TAZ endogenous targets *CTGF* and *CYR61* in A549- and Calu-1 cell-overexpressed lncRNA Control or lncRNA CRYBG3 after treatment with the indicated siRNA. **F** Luciferase assays in Calu-1 cells transfected with 8 × GTIIC-Lux reporter and treated as indicated. **G** Representative immunofluorescence images of YAP/TAZ (green) and phalloidin (red). Scale bars, 20 μm. **H** Quantifications of YAP/TAZ localization in A549 cells as indicated treatments. All the experiments are repeated three independent times and data are presented as mean ± s.d. *P-*values were calculated by unpaired Student’s *t*-test. *p < 0.05; **p < 0.01; ***p < 0.001; ****p < 0.0001.

The Hippo pathway, especially its core unit LATS1/2, is a well-characterized kinase cascade that phosphorylates YAP/TAZ, leading to cytoplasmic retention of YAP and TAZ protein degradation [12, 39]. In this study, we examined the effect of overexpression of lncRNA CRYBG3 on YAP/TAZ and LATS1/2 phosphorylation. We found that lncRNA CRYBG3 expression did not affect the total LATS1/2 protein levels but significantly increased the levels of phosphorylated p-LATS1/2 compared with the control condition (Fig. 6A). In line with LATS1/2 phosphorylation, lncRNA CRYBG3 also induced YAP phosphorylation on Ser127 (Fig. 6A, the original western blots are in Supplemental Material 2). Similarly, lncRNA CRYBG3 blocked TAZ protein levels (Fig. S4G, the original western blots are in Supplemental Material 3). This result implies that lncRNA CRYBG3 might activate LATS1/2 to restrain YAP/TAZ activity. We tested this hypothesis by knocking down LATS1/2 using siRNA in cells overexpressing lncRNA CRYBG3. We evaluated the expression levels of direct YAP/TAZ target genes, such as *CTGF* and *CYR61*, as a proxy of the transcriptional activity of YAP/TAZ. The result in two different cell lines with independent siRNAs showed that knockdown of LATS1/2 could restore the lncRNA CRYBG3-mediated inhibition of YAP/TAZ transcriptional activity (Fig. 6B–E, verification in Fig. S4H, I, J, the original western blots are in Supplemental Material 4). Similar results were obtained from the luciferase-reporter assays (Fig. 6F and Fig. S4K). Immunofluorescence shows that depleting LATS1/2 could restore YAP/TAZ nuclear entry without affecting actin organization (Fig. 6G, H, and Fig. S4L). These results indicate that lncRNA CRYBG3 mediates the cytoplasmic retention of YAP/TAZ and reduces its transcriptional activity through phosphorylation by LATS1/2.

## Discussion

The findings presented here demonstrate that ionizing radiation leads to a severe reduction of tumor-tissue stiffness that is mediated by IR-induced expression of lncRNA CRYBG3. Indeed, overexpression of lncRNA CRYBG3 impaired the cytoskeleton organization and mechanotransduction pathway phenocopying the effects of IR exposure. Our data suggest that lncRNA CRYBG3 induced by ionizing radiation impairs the mechanosignaling pathway, thereby regulating tumor growth and metastasis through the activity of mechanotransduction effector molecules YAP/TAZ. Furthermore, lncRNA CRYBG3 exerted a tumor-suppressor function by inhibiting the proliferation, invasion, and metastasis of lung cancer cells through YAP/TAZ.

LncRNAs are endogenously transcribed RNA molecules and are generally considered as noncoding (some literature reports that they can also encode functional short peptides) [40]. LncRNAs can regulate multicellular functions such as chromatin remodeling, gene editing, promotion/inhibition of transcription, and translation. Mounting evidence suggests that lncRNAs are mutated or dysregulated in a variety of human pathologies, especially tumorigenesis [41]. LncRNA GAS5, as a tumor suppressor, induces the upregulation of Plexin C1 by decreasing miR-222 levels in human glioma cells, thereby inducing cofilin inactivation and promoting cell migration and invasion [42]. lncRNA SNHG5 acts as a sponge of miR-26a and competitively binds ROCK with miR-26a to promote the proliferation, invasion, and migration of osteosarcoma cells [43]. Also, our previous work established that lncRNAs can regulate cancer progression and cell proliferation through their direct modulation of cytoskeletal structure [30]. All these studies point toward the common theme, that of lncRNAs interfere with the cytoskeleton in one way or another, but the link between lncRNAs and cellular mechanotransduction in the context of cancer progression has not yet been elucidated. In this study, we manipulated F-actin organization either by treatment with jasplakinolide (Fig. 4), a commonly used drug that potently induces actin polymerization, also stabilizing pre-existing actin filaments or by interference with ADF/CFL1 (Fig. 5), an endogenously F-actin organizer, to alleviate lncRNA CRYBG3-mediated repression of YAP/TAZ transcriptional activity. Our work represents the first direct evidence that lncRNA CRYBG3 regulates YAP and TAZ subcellular localization and transcriptional activity by modulating the mechanotransduction pathway.

To date, there have been no prior investigations addressed on how irradiation affects the mechanical property of the tumor tissue or cellular mechanotransduction. We observed a striking decrease in tumor stiffness early after irradiation. This was paralleled by a decrease in the thickness of collagen fibers (Fig. S1A), which are critical components of the tumor ECM, and phosphorylation of MLC2 (Fig. 1B), which regulates the contraction of actin. It is thus tempting to speculate that irradiation decreased tumor stiffness by affecting both ECM structure and the tensional state of the cancer cells themselves.

Here, we have uncovered the modulation of cellular mechanotransduction as a previously underappreciated effect of radiation therapy. This study exemplifies the conceptual importance of mechanotransduction in understanding the biological effects of radiation therapy and, vice versa, of ionizing radiation in mechanotransduction research. If broadly applicable, this would expand manifolds the influence of mechanotransduction to the biology field that is well established for mechanobiology-research study, but so far largely overlooked in the radiation-research field. Potential topics of interest where mechanobiology and radiation research might intersect are cell cycle arrest, aging, and apoptosis. This study also highlights the need to adopt a wider interpretative lens and focus for the burgeoning field of YAP/TAZ mechanobiology. In sum, our research suggests the existence of a regulatory mechanism that can be summed up as follows: “Ionizing Radiation → LncRNA CRYBG3 → ADF/CFL1 --- |F-actin --- | LATS1/2 --- | YAP/TAZ → tumor proliferation, migration and invasion”. Finally, our research highlights lncRNA CRYBG3 as a potential new target for tumor diagnosis and treatment.

## Supplementary information

supplementary figure legends

Figure S1

Figure S2

Figure S3

Figure S4

Supplemental Material

aj-checklist

## Data Availability

The datasets generated and/or analyzed during the current study are available from the corresponding author on reasonable request.
