# Supplementary material for: Ionizing radiation-induced long noncoding RNA CRYBG3 regulates YAP/TAZ through mechanotransduction
Source: Cell Death Dis. 2022 Mar 4;13(3):209. doi: 10.1038/s41419-022-04650-x (PMC8897501; doi:10.1038/s41419-022-04650-x)
Supplement: Supplementary file 1 — supplementary figure legends [file 41419_2022_4650_MOESM1_ESM.pdf]

Supplementary Information

Figure S1.

A Representative sirius red stain images of collagen in xenografted tumors with or without X-ray exposure. Scale bars, 50 μm. **B** Expression of lncRNA CRYBG3 in response to multiple ionizing radiation.

Figure S2.

A Heatmap showing the changes of gene expression in lncRNA CRYBG3 (LNC CRYBG3) overexpressed A549 cells compared with lncRNA Control (LNC Control). **B** Volcano plot showing the altered genes in lncRNA CRYBG3 overexpressed cells compared with lncRNA Control. **C** Venn diagram showing the intersection of YAP/TAZ direct target genes with genes set which collect from fig. S2A. **D** Table of log2 Fold Change (log2FC) in the intersection of YAP/TAZ target genes with differential genes in overexpressed lncRNA CRYBG3 cells compared with lncRNA Control. **E** Immunofluorescence analysis determined YAP/TAZ localization and the microfilaments morphology in A549 cells. Scale bars, 20 μm. **F** The quantifications of nuclear YAP/TAZ ratio from Fig. 2D and Fig. S2E. **G** qRT-PCRs assessing the expression levels of the YAP/TAZ endogenous targets *CTGF*, *ANKRD1* and *CYR61* in A549 cells. **H** qRT-PCRs assessing the expression levels of *lncRNA CRYBG3* in A549 and

Calu-1 cells treated with a gradient concentration of lncRNA CRYBG3. **I** Luciferase assays in A549 cells transfected with a synthetic reporter for YAP-TEAD-dependent transcription (8xGTIIC-Lux). **J** qRT-PCRs assessing the expression levels of *lncRNA CRYBG3* in A549 cells transfected with lncRNA CRYBG3 shRNA or control shRNA. **K** qRT-PCRs assessing the expression levels of the YAP/TAZ endogenous targets *CYR61* in A549 cells exposed to 4 Gy X-rays (IR group) or control (control group).

Figure S3.

A, **B** qRT-PCRs assessing the *lncRNA CRYBG3* and *TAZ* expression levels with the indicated shRNAs. **C** qRT-PCRs assessing the expression levels of *CTGF* with the indicated shRNAs. **D** Representative HE staining images of the metastatic nodules of the lungs as indicated treatment. Scales as indicated.

Figure S4.

A, **B** qRT-PCRs assessing the expression levels of *lncRNA CRYBG3* in A549 and Calu-1 cells as indicated treatments. **C**, **D** qRT-PCRs assessing the expression levels of *ADF* and *CFL1* in A549 cells overexpressed lncRNA Control (LNC Control) or lncRNA CRYBG3 (LNC CRYBG3). **E**,

F qRT-PCRs assessing the expression levels of *LncRNA CRYBG3* in A549 and Calu-1 cells overexpressed lncRNA Control or lncRNA CRYBG3 after treated with the indicated siRNA. **G** Western blot analysis of TAZ proteins in A549 cells transfected with lncRNA Control or lncRNA CRYBG3. **H** Western blot analysis of LATS1 proteins in A549 cells overexpressed lncRNA Control or lncRNA CRYBG3 after treated with the indicated siRNA. **I**, **J** qRT-PCRs assessing the expression levels of *lncRNA CRYBG3* in A549 and Calu-1 cells overexpressed lncRNA Control or lncRNA CRYBG3 after treated with the indicated siRNA. **K** Luciferase assays in A549 cells transfected with 8×GTIIC-Lux reporter and as indicated treatments. **L** Representative immunofluorescence images of YAP/TAZ (green) and phalloidin (red) in Calu-1 cells as indicated treatments. Scale bars, 20 μm.
