# Supplementary figures and images for: Ionizing radiation-induced long noncoding RNA CRYBG3 regulates YAP/TAZ through mechanotransduction
Source: Cell Death Dis. 2022 Mar 4;13(3):209. doi: 10.1038/s41419-022-04650-x (PMC8897501; doi:10.1038/s41419-022-04650-x)

Figure S1

A

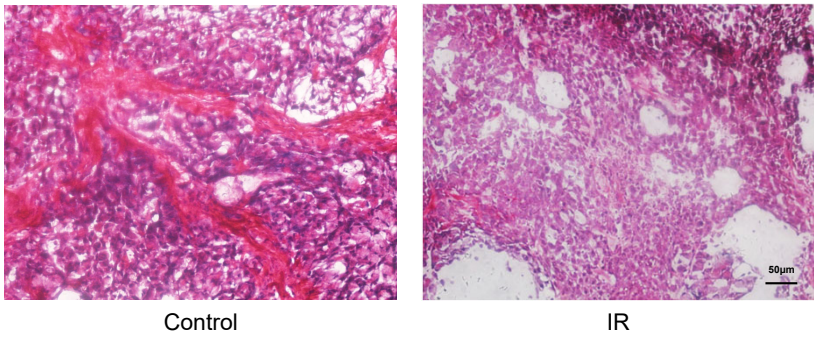

B

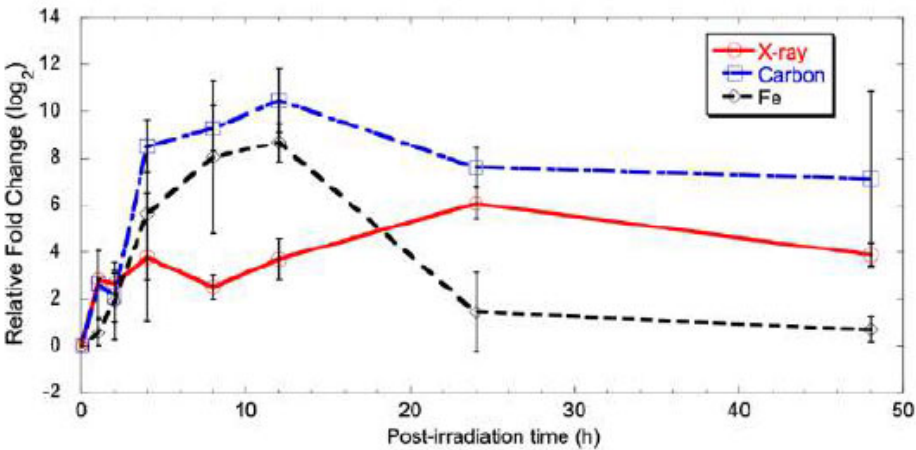

Supplement: Supplementary file 2 — Figure S1 [file 41419_2022_4650_MOESM2_ESM.pdf]

Figure S2

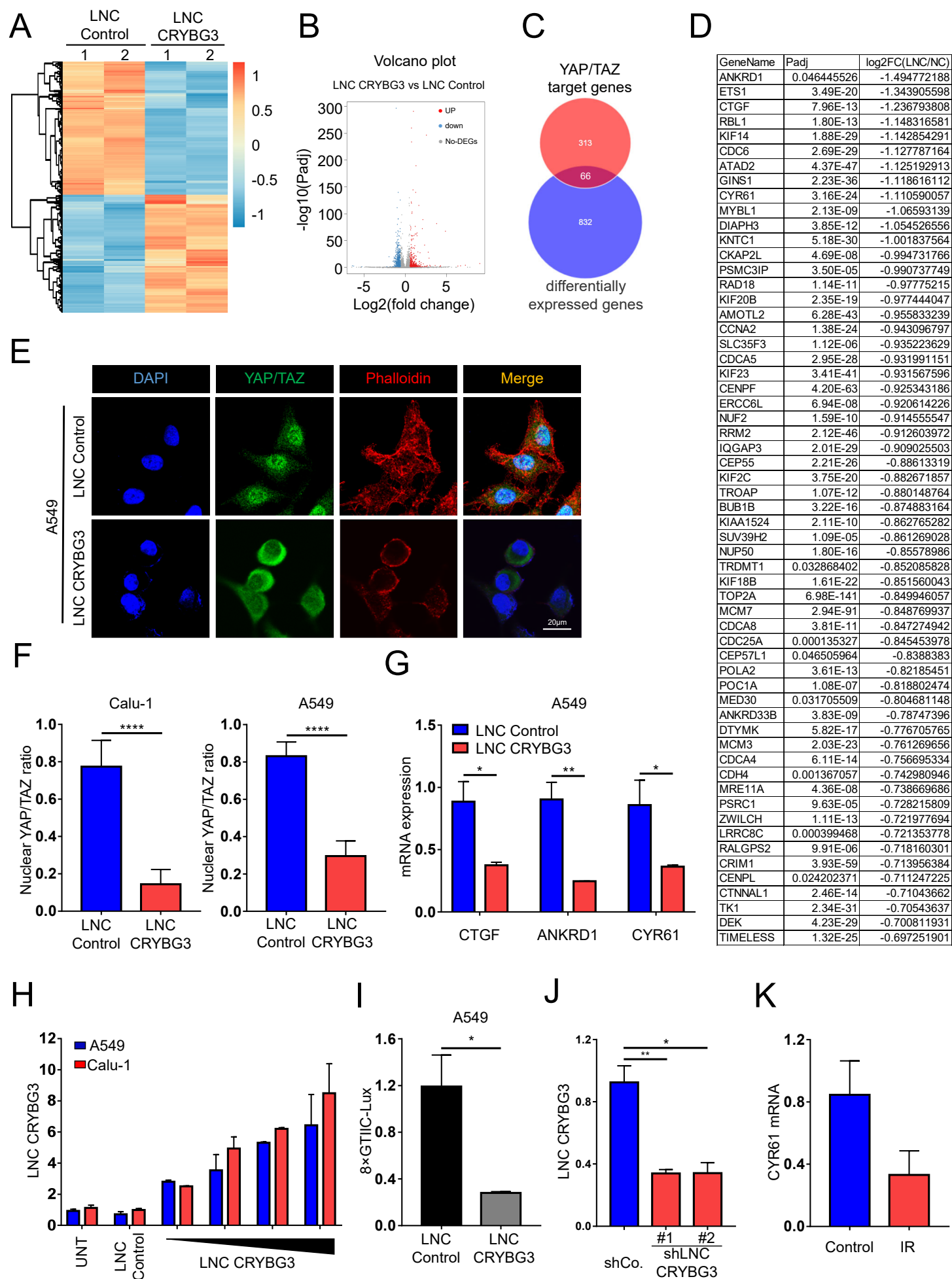

Supplement: Supplementary file 3 — Figure S2 [file 41419_2022_4650_MOESM3_ESM.pdf]

Figure S3

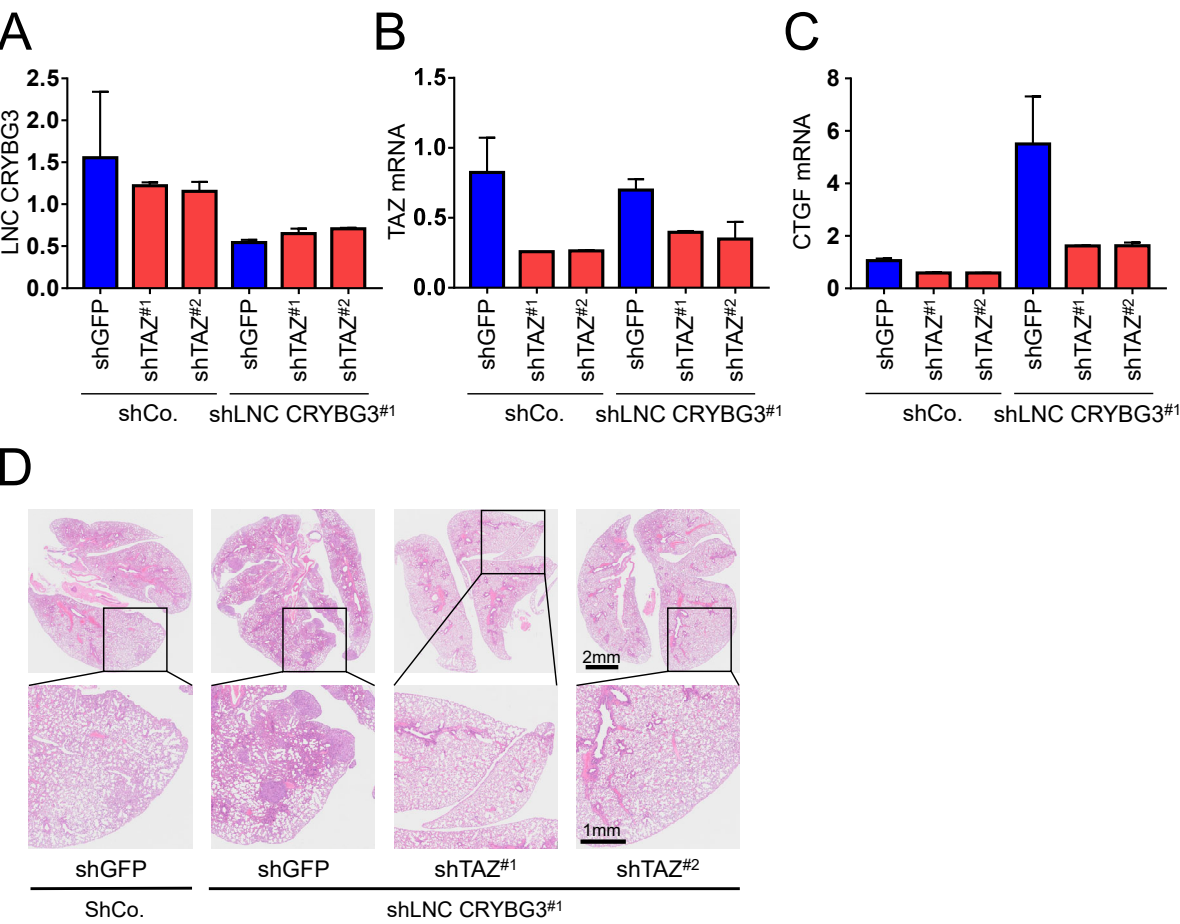

Supplement: Supplementary file 4 — Figure S3 [file 41419_2022_4650_MOESM4_ESM.pdf]

Figure S4

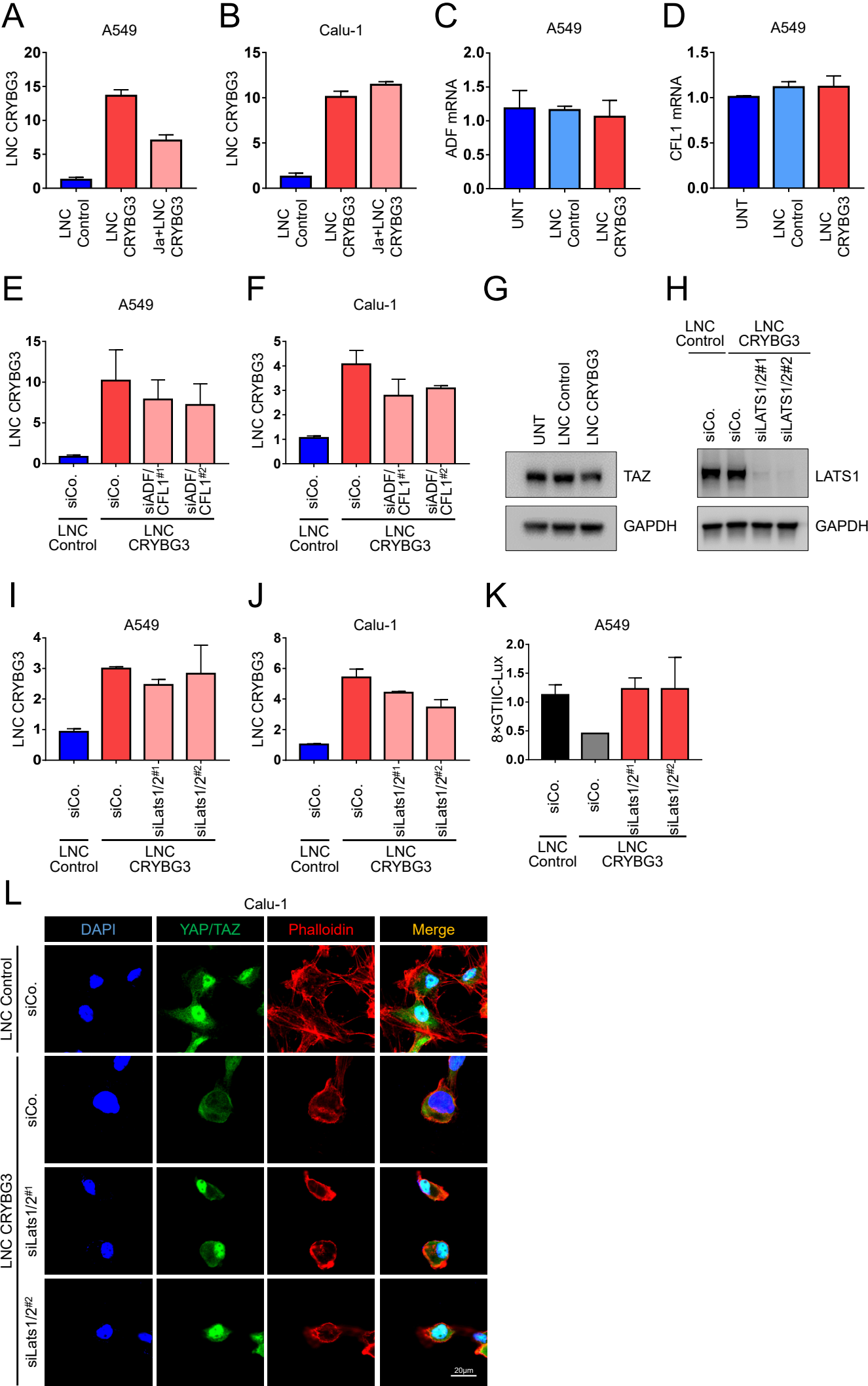

Supplement: Supplementary file 5 — Figure S4 [file 41419_2022_4650_MOESM5_ESM.pdf]

Figure 2E

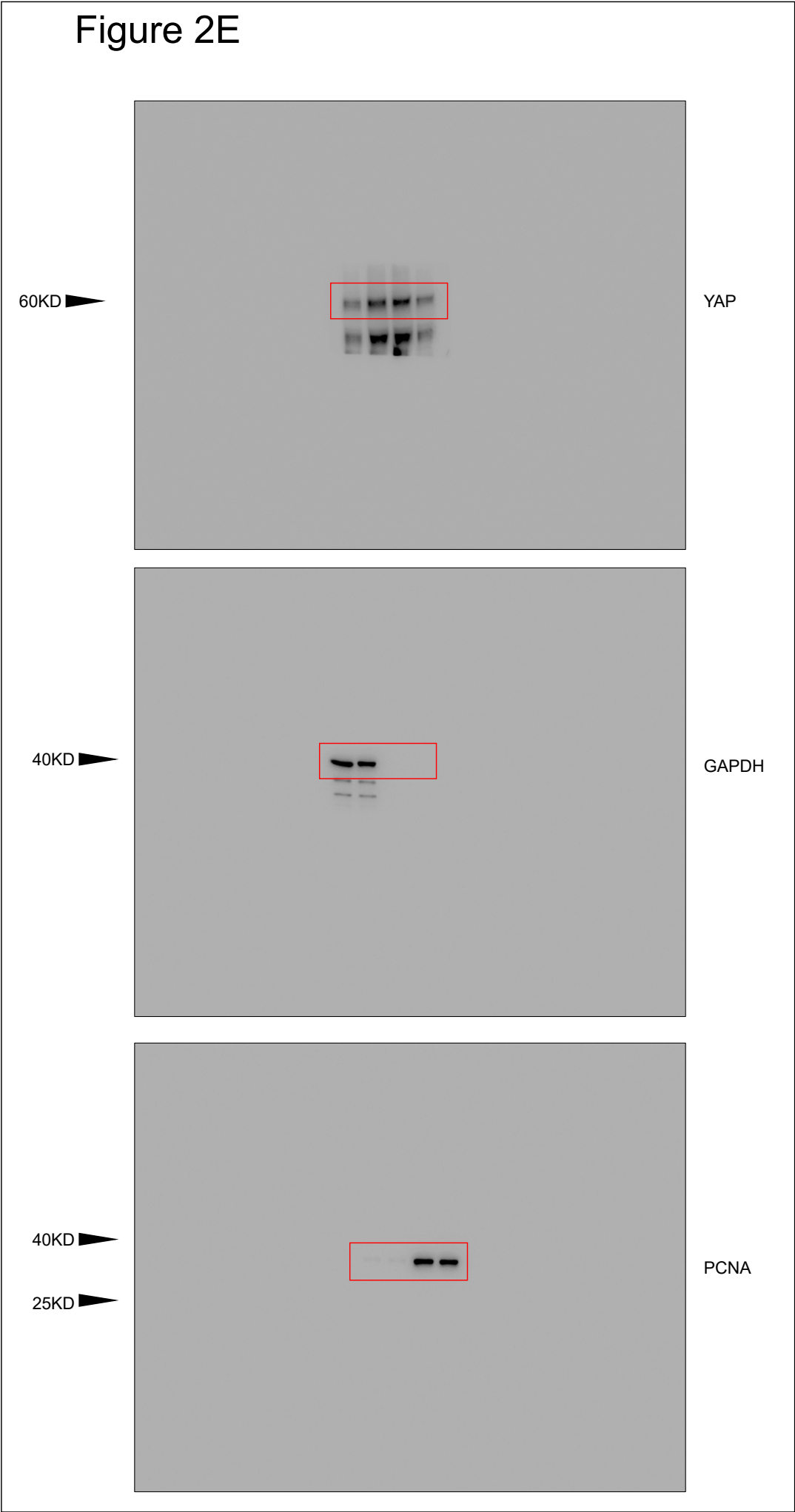

Figure 6A

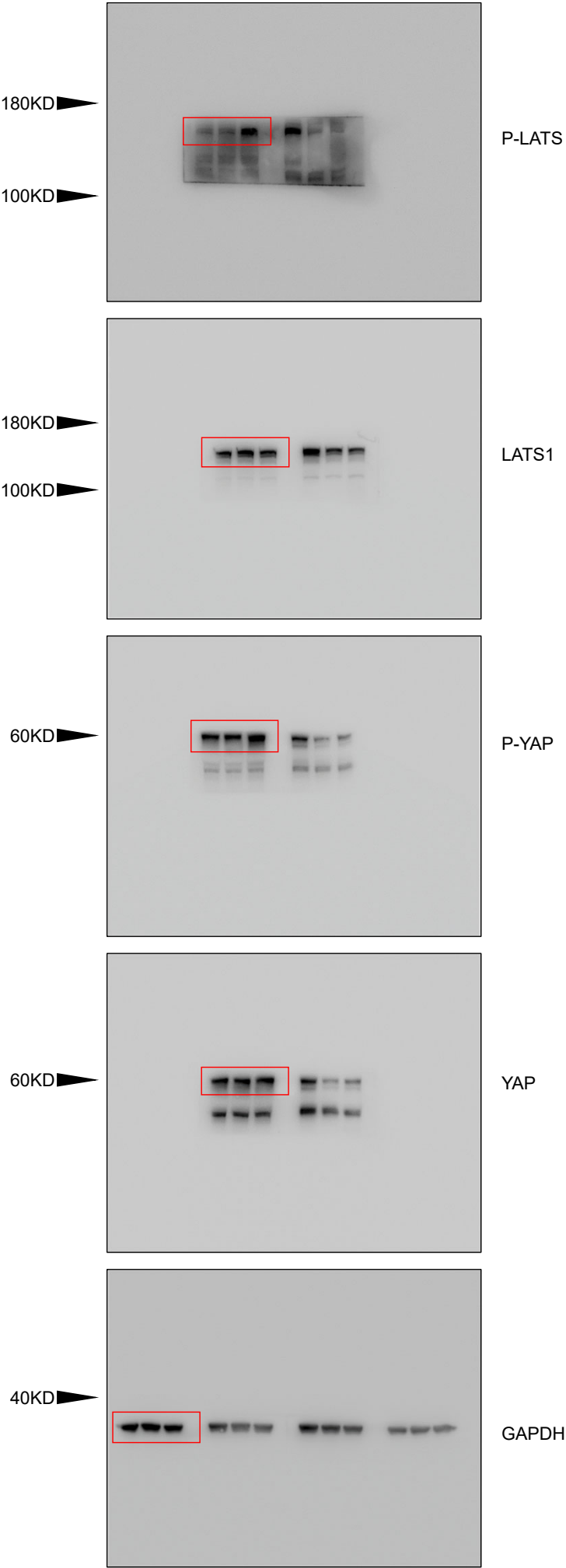

Figure S4G

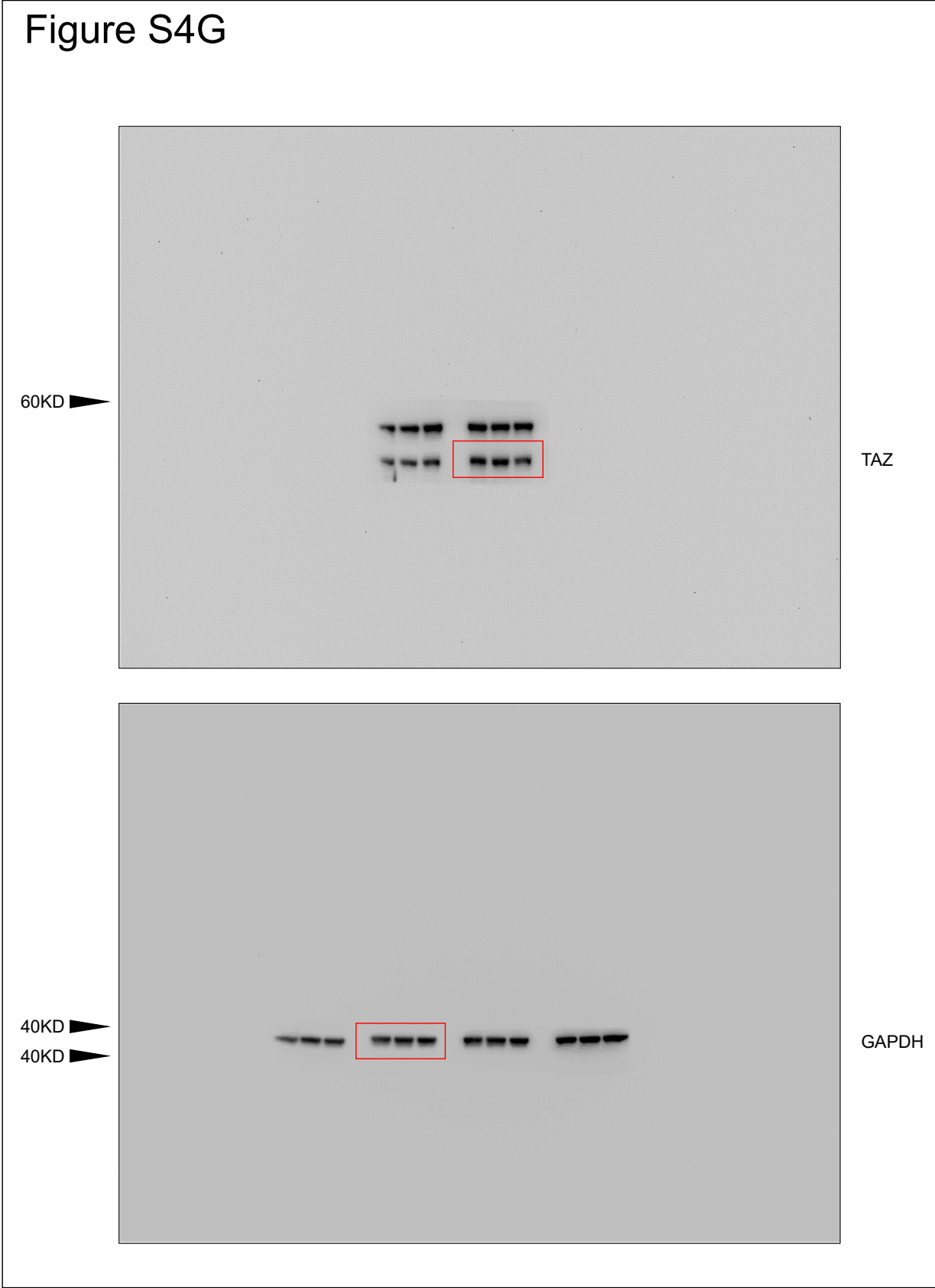

Figure S4H

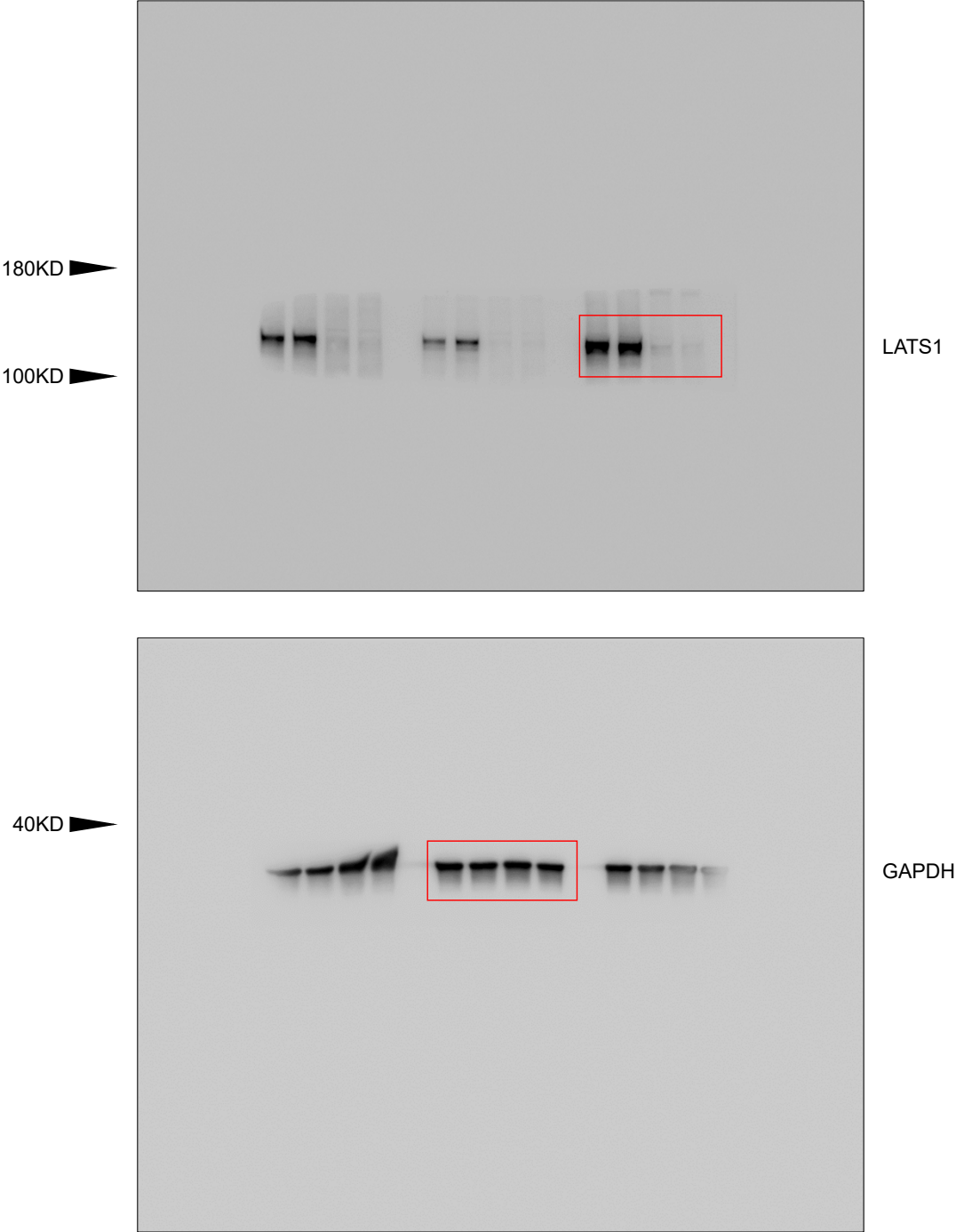

Supplement: Supplementary file 6 — Supplemental Material [file 41419_2022_4650_MOESM6_ESM.pdf]
